# Patient reported experiences of health care, quality of life and preoperative information in colon cancer

**DOI:** 10.2340/1651-226X.2024.40933

**Published:** 2024-11-14

**Authors:** Maria Reinwalds, Charlotta Larsson, Rode Grönkvist, Eva Angenete

**Affiliations:** a Department of Surgery, SSORG – Scandinavian Surgical Outcomes Research Group, Institute of Clinical Sciences, Sahlgrenska Academy, University of Gothenburg, Gothenburg, Sweden; b Region Västra Götaland, Sahlgrenska University Hospital/Östra, Department of Surgery, Gothenburg, Sweden; c School of Public Health and Community Medicine, Institute of Medicine, Sahlgrenska Academy, University of Gothenburg, Gothenburg, Sweden

**Keywords:** colonic neoplasm, quality of life, health care encounters, patient reported experiences, PREM, questionnaire

## Abstract

**Background and purpose:**

Cancer may create problems and needs associated with impaired quality of life (QoL). The first health care encounter is important to enable patients to cope and may ultimately impact QoL. The aim of this study was to describe the patients’ experiences of encounters with health care professionals. Another aim was to explore the possible impact that the encounters may have on QoL 1 year after a colon cancer diagnosis. We also wanted to investigate whether patients had received information about treatment related side-effects.

**Patients and methods:**

This substudy within the QoLiCOL (Quality of Life in COLon cancer) study included 1687 patients (male *n* = 876, female *n* = 811, mean age 71) between 2015 and 2019. Questionnaires were answered at diagnosis and after 1 year. QoL was self-assessed with a seven-point Likert scale. Analyses were performed using descriptive statistics and ordinal logistic regression.

**Results:**

A total of 1,550 patients (91.9%) reported feeling well received by health care professionals. We found no statistically significant association with QoL. Patients (87%) reported feeling well informed about their treatment, but few patients recalled having received information regarding potential side effects on bowel or sexual function.

**Interpretation:**

Patients with colon cancer generally had a positive experience of the encounter with health care where they felt both well received and well informed. However, the amount of relevant information received was scarce. This indicates that it may be difficult to identify whether patients are properly informed prior to treatment for colon cancer only by asking if they feel well informed.

## Introduction

The primary treatment for colon cancer is surgery followed in some cases by adjuvant chemotherapy. Survival has improved over the last decades and 5-year survival is over 60% in Sweden [1].

A cancer diagnosis affects patients in many ways and may create problems and needs changeable over time [2, 3]. Unmet problems and needs are associated with impaired quality of life (QoL) [3–5]. During the last decades, increasing consideration has been given to overall QoL and not only to disease-free and long-term survival. A Swedish study reported that a considerable number of patients operated for colorectal cancer have impaired global QoL with a significant association with anxiety and several physical symptoms [6].

The European Society for Medical Oncology and the World Health Organization advocate for a more person-centred care [2, 7]. One core element of person-centred care is initiating a partnership between patient and health care professionals at the first encounter. Thus, the first meeting is not only about receiving a cancer diagnosis but also to establish a partnership [8, 9].

Research has found that a trusting relationship between patient and physician decreased the patient’s fears and facilitated shared decision making and communication [10, 11]. It has been suggested that there is an association between the patient’s trust in their physician and changes in physical function [12]. This underlines the importance of establishing a trustful and close relationship between physician and patient, to enable patients to cope and ultimately impact health- related QoL. Providing sufficient information about the disease, treatment and recovery process in a clear and individualized way is also important to help patients cope [13, 14]. A trusting patient-provider relationship is associated with better adherence to treatment and surveillance indicating that this could potentially increase survival [10, 15].

Studies have shown that long waiting times for results, tests or in the diagnostic pathway caused dissatisfaction and feelings of being ignored and neglected [4, 16–18], which may have a negative effect on QoL [4, 19]. However, one study indicated that the negative effect could potentially be alleviated by experiences of positive communication with health care professionals [16].

The relationship between patient and health care professionals, initiated at the first health care encounter, could be of importance to patients’ cancer trajectory, potentially affecting QoL. This has to our knowledge not been explored in large studies among patients with colon cancer. In this cohort study (QoLiCOL) we have not only created a new questionnaire with a clinimetric approach but also included psychometrically validated instruments. We have explored the patients’ experiences during and after colon cancer diagnosis and treatment.

The aim of this study was to quantitatively describe the patients’ experience of encounters with health care professionals. Another aim was to explore the possible impact that the care encounters may have on QoL 1 year after a colon cancer diagnosis. We hypothesized that a negative experience of care encounters was associated with low QoL. We also wanted to investigate whether patients had received information about side-effects that could arise from their treatment.

## Patients and methods

### Study design

The QoLiCOL (Quality of Life in COLon cancer) study was a prospective, non-interventional, longitudinal multicentre cohort study according to the STROBE guidelines exploring physical symptoms, functional outcomes, socioeconomic burden and health-related QoL among patients with colon cancer from diagnosis, during treatment and up to 3 years after diagnosis. The study included 1,891 patients from 21 hospitals in Denmark and Sweden between 2015 and 2019. Patients were eligible for participation if newly diagnosed with colon cancer and a written informed consent was obtained. Patients unable to understand or read Swedish or Danish and patients below the age of 18 were not included. Questionnaires were distributed by a neutral third party, and not the treating surgical clinic, at diagnosis and after 1 and 3 years [20]. Clinical data were retrieved from the validated Swedish and Danish national quality registers for colon cancer [21, 22].

Sixteen in-depth qualitative interviews were held with patients with colon cancer and then subjected to qualitative content analysis, resulting in themes. These themes were used to construct questions. The questionnaires were based on a clinimetric approach [23] and were constructed and validated according to a previously published method [24]. Content validation and item selection were performed by an expert panel consisting of oncologists, anaesthesiologists, colorectal surgeons, gynaecologists and nurses specialized in surgery. The questions were face validated by patients diagnosed with colon cancer as previously described [25, 26]. The questionnaires contained over 200 detailed questions on patients’ reflections on illness, health and well-being, experiences of health care, psychological and physical function, and rehabilitation. They also included previously validated generic instruments such as the 29-item Sence of Coherence scale (SOC-29) [27].

This study is based on data collected from the questionnaires at baseline (at diagnosis) and after 1 year. Patients who had answered at least one questionnaire were included.

### Outcome measures

The primary outcome in this study was the association of patient reported experience of health care at baseline with QoL at 1-year follow-up. Experience of health care was assessed at baseline with the question ‘Have you felt well received in the surgical clinic?’ with the answering alternatives ‘No’, ‘Yes, somewhat’, ‘Yes, moderately’ and ‘Yes, very’. QoL was self-reported by the patients by answering the question: ‘How would you describe your QoL during the last month?’, with a seven-point Likert scale (worst possible QoL = 0, Best possible QoL = 6)[25]. Secondary outcomes were the association of several other aspects of the health care encounter at baseline with QoL at 1 year follow-up. All questions assessed are presented in Table 1.

**Table 1:** Descriptive data of patient reported experience of health care encounters.

	Overall (*N* = 1,687) (%)
**Have you felt well received in the surgical clinic?**	
No	2 (0.1)
Yes, somewhat	9 (0.5)
Yes, moderately	65 (3.9)
Yes, very	1476 (87.5)
Missing	135 (8.0)
**Do you feel that you have been taken seriously in the surgical clinic?**	
No	1 (0.1)
Yes, somewhat	7 (0.4)
Yes, moderately	50 (3.0)
Yes, very	1494 (88.6)
Missing	135 (8.0)
**Have you felt that you are in safe hands in the surgical clinic?**	
No	2 (0.1)
Yes, somewhat	16 (0.9)
Yes, moderately	103 (6.1)
Yes, very	1428 (84.6)
Missing	138 (8.2)
**Have you – during the diagnostic process- experienced reasonable waiting times for colonoscopy, x-ray, follow-up visits etc?**	
No	191 (11.3)
Yes, somewhat	119 (7.1)
Yes, moderately	374 (22.2)
Yes, very	858 (50.9)
Missing	145 (8.6)
**Did you dare to ask the questions you wanted to the doctor in the surgical clinic?**	
No	33 (2.0)
Yes	1513 (89.7)
Missing	141 (8.4)
**Did you have the opportunity to ask the doctor the questions you wanted in the surgical clinic?**	
No	22 (1.3)
Yes	1524 (90.3)
Missing	141 (8.4)

Variable labels used in regression analyses are underlined.

### Statistical analysis

Prior to data analysis a detailed statistical analysis plan was developed. No power calculation was performed as this was a secondary outcome of the QoLiCOL study and the analysis was considered exploratory. The patient reported experience of health care encounters and information received are presented using descriptive statistics. To explore if patient reported negative experience of being encountered by health care professionals was associated with low QoL ordinal logistic regression was used. To assess whether the proportional odds assumption was tenable for the model, a series of binary logistic regression models was fitted, each with a different cut point in 1-year QoL. Based on previous research [28] and clinical experience a directed acyclic graph was constructed to identify confounding. The following covariates were included and adjusted for in the statistical models: age, sex, education, ASA classification, intention of treatment, recurrent disease, adjuvant chemotherapy, alcohol intake, depression, pain, marital status, sense of coherence and negative intrusive thoughts as well as baseline QoL. All analyses were multivariate. The hypotheses were tested at a 5% significance level. Missing responses to the SOC-29 questionnaire were imputed in the following way: if less than 25% of responses were missing, the missing values were imputed as the median of the answered questions for computation of SOC-29 score. If more than 25% of responses were missing, the SOC-29 score was considered missing [28].

Secondary outcomes were assessed similarly to the primary outcome, using an ordinal logistic regression model with self-reported QoL at 1 year as the outcome. All secondary outcomes (see Table 1) were included in the model as independent variables.

Missing data were handled using multiple imputation by chained equations [29]. This imputation was carried out using the ‘mice’ package version 3.16.0 in R [30]. Analyses were performed using R version 4.3.3 [31].

### Ethical aspects

The QoLiCOL study was approved by the Regional Ethical Committee in Gothenburg (Dnr 957-14). The study was registered at clinicaltrials.gov (NCT02530593).

## Results

**Figure 1:**
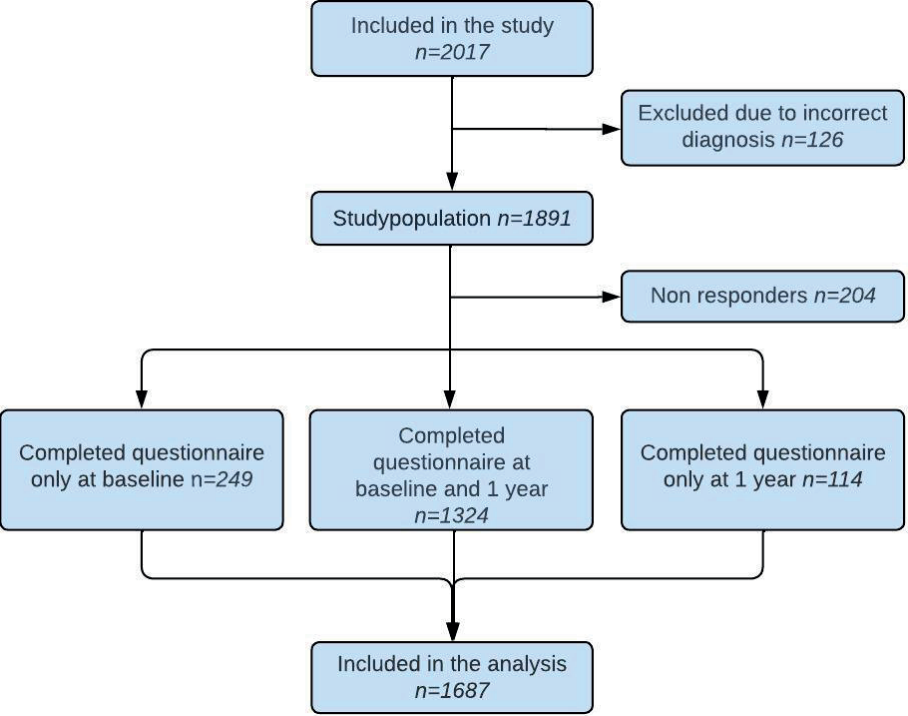
Flow chart of patients.

The QoLiCOL study population consists of 1,891 patients included between May 2015 and September 2019. Of these, 1,687 (89%) patients completed at least one of the questionnaires, at baseline or after 1 year, and were included in the analyses (Figure 1). Patient demographics are presented in Table 2. The mean age was 71 years, 51.9 % were male.

**Table 2:** Demography of the study population.

	Overall (*N* = 1,687) (%)
**Sex**	
Male	876 (51.9)
Female	811 (48.1)
**Age (years)**	
Mean (SD)	71 (10)
**Education**	
No university education	1097 (65.0)
University education	440 (26.1)
Missing	150 (8.9)
**Primary occupation**	
Working	260 (15.4)
Sick leave	69 (4.1)
Retired	1176 (69.7)
Other	31 (1.8)
Missing	151 (9.0)
**Relationship status**	
No relationship	456 (27.0)
In a relationship	1090 (64.6)
Missing	141 (8.4)
**Smoker (yes/no)**	
No	1475 (87.4)
Yes	77 (4.6)
Missing	135 (8.0)
**Risk consumption of alcohol (yes/no)**	
No	1353 (80.2)
Yes	102 (6.0)
Missing	232 (13.8)
**Physical activity (Saltin-Grimby)**	
1	266 (15.8)
2	1062 (63.0)
3	206 (12.2)
4	12 (0.7)
Missing	141 (8.4)
**Self-reported depression**	
No	1303 (77.2)
Yes	255 (15.1)
Missing	129 (7.6)
**Sense of coherence (SOC-29)**	
Mean (SD)	150 (19)
Missing	144 (8.5)
**Negative intrusive thoughts**	
None	256 (15.2)
Less than daily	857 (50.8)
At least daily	436 (25.8)
Missing	138 (8.2)
**Tumour stage cT**	
T1–2	448 (26.6)
T3	655 (38.8)
T4	275 (16.3)
TX	265 (15.7)
Missing	44 (2.6)
**Tumour stage cN**	
N0	872 (51.7)
N1–2	677 (40.1)
NX	98 (5.8)
Missing	40 (2.4)
**Tumour stage cM**	
M0	1493 (88.5)
M1	153 (9.1)
Missing	41 (2.4)
**Comorbidity (ASA-classification)**	
1	196 (11.6)
2	932 (55.2)
3	407 (24.1)
4	27 (1.6)
5	1 (0.1)
Missing	124 (7.4)
**Intention of treatment**	
Curative	1496 (88.7)
Palliative	12 (0.7)
Unknown	179 (10.6)
Quality of life at baseline*	
0	6 (0.4)
1	41 (2.4)
2	211 (12.5)
3	373 (22.1)
4	459 (27.2)
5	345 (20.5)
6	110 (6.5)
Missing	142 (8.4)
Quality of life at 1-year follow-up*	
0	3 (0.2)
1	13 (0.8)
2	60 (3.6)
3	189 (11.2)
4	393 (23.3)
5	525 (31.1)
6	234 (13.9)
Missing	270 (16.0)

* QoL was self-reported by the patients by answering the question: ‘How would you describe your quality of life during the last month?’, with a seven-point Likert scale (worst possible QoL = 0, Best possible QoL = 6).

Non-responders showed similar distribution of sex, age, and intention of treatment as patients included, but had higher ASA classification and slightly more advanced tumour stage. (Supplementary Table 1).

Our primary outcome was measured with the question ‘Have you felt well received in the surgical clinic?’. The most frequent answer (88%) was ‘Yes, very’. Only two patients reported not feeling well received. Feeling well received had no significant association with QoL (OR = 0.99, 95%CI 0.65–1.5, *p* = 0.97). However, there were significant associations between QoL at 1 year and ASA classification (OR = 0.77, 95% CI 0.66–0.91, *p* < 0.01), recurrence at 1 year (OR = 0.28, 95% CI 0.18–0.44, *p* < 0.0001), sense of coherence (OR = 1.03, 95% CI 1.03–1.04, *p* < 0.0001), daily negative intrusive thoughts (OR = 0.68, 95% CI 0.47–0.98, *p* < 0.04) and QoL at baseline (OR = 1.47, 95% CI 1.33–1.63, *p* < 0.0001) (Table 3). Only one patient felt not being taken seriously and only two patients felt they were not in safe hands at the surgical clinic. Thirty-three patients (2%) did not dare to ask the questions they wanted to and 22 patients (1.3%) felt they did not get the opportunity to ask the questions they wanted to. A total of 191 patients (11.3%) reported that they experienced the waiting time as unreasonable. No significant association with QoL was found in any of the secondary endpoints (Table 4).

**Table 3:** Primary analysis – Multiple ordinal logistic regression with QoL at 1 year as outcome.

Covariate	Estimate (OR)	CI (2.5%)	CI (97.5%)	*p*
Well received	0.99	0.65	1.50	0.97
Age	1.01	1.00	1.02	0.20
Sex (Female)	1.01	0.83	1.24	0.90
Education (University)	0.92	0.73	1.16	0.46
In a relationship	0.96	0.76	1.21	0.73
Risk consumption of alcohol	1.28	0.86	1.90	0.23
Self-reported depression	0.87	0.62	1.21	0.40
Chronic pain	0.70	0.48	1.00	0.05
Sense of coherence (SOC-29 score)	1.03	1.03	1.04	<0.001
Negative intrusive thoughts (occurring but less than daily)	0.74	0.54	1.03	0.07
Negative intrusive thoughts (at least daily)	0.68	0.47	0.98	0.04
Quality of life (at baseline)	1.47	1.33	1.63	<0.001
ASA-classification	0.77	0.66	0.91	<0.001
Palliative intention of treatment	1.34	0.40	4.47	0.63
Unknown intention of treatment	1.09	0.77	1.54	0.63
Recurrence at 1 year	0.28	0.18	0.44	<0.001
	1.04	0.83	1.31	0.71

Adjuvant chemotherapy

**Table 4:** Secondary analysis – Multiple ordinal logistic regression with QoL at 1 year as outcome.

Covariate	Estimate (OR)	CI (2.5%)	CI (97.5%)	*p*
Taken seriously	0.84	0.47	1.50	0.55
In safe hands	1.04	0.71	1.51	0.86
Reasonable waiting times	1.01	0.91	1.12	0.88
Dare to ask	0.57	0.22	1.47	0.24
Opportunity to ask	1.47	0.57	3.80	0.42
Age	1.01	1.00	1.02	0.21
Sex (Female)	1.01	0.82	1.24	0.91
Education (University)	0.91	0.72	1.15	0.43
In a relationship	0.96	0.76	1.21	0.74
Risk consumption of alcohol	1.29	0.86	1.93	0.21
Self-reported depression	0.86	0.62	1.20	0.38
Chronic pain	0.68	0.47	0.98	0.04
Sense of coherence (SOC-29 score)	1.03	1.03	1.04	<0.001
Negative intrusive thoughts (occurring but less than daily)	0.75	0.54	1.04	0.08
Negative intrusive thoughts (at least daily)	0.68	0.47	0.99	0.04
Quality of life (at baseline)	1.47	1.33	1.63	<0.001
ASA-classification	0.77	0.65	0.90	<0.001
Palliative intention of treatment	1.38	0.42	4.57	0.59
Unknown intention of treatment	1.07	0.76	1.51	0.69
Recurrence at 1 year	0.29	0.18	0.45	<0.001
Adjuvant chemotherapy	1.04	0.83	1.30	0.74

Most patients (87%) reported feeling well informed about their cancer disease and the planned treatment. However, only 51% felt that they were informed about potential side-effects on bowel function, and this was even lower for sexual dysfunction, only 3.7%. Patients also experienced a lack of information regarding dietary advice or recommended exercise and physical activity (Table 5).

**Table 5:** Descriptive data of patient reported experience of received information

	Overall(N=1687)
**Do you feel well-informed about your cancer diagnosis and the planned treatment?**	
Yes	1472 (87.3%)
No	64 (3.8%)
Missing	151 (9.0%)
**Have you received information from your doctor about unwanted side effects of the treatment regarding bowel function?**	
Yes	860 (51.0%)
No	646 (38.3%)
Missing	181 (10.7%)
**Have you been informed of where to turn to if you have unwanted side effects?**	
Yes	788 (46.7%)
No	697 (41.3%)
Missing	202 (12.0%)
**Have you and your doctor discussed your upcoming treatment’s possible effects on your sex life?**	
Yes	63 (3.7%)
No	1410 (83.6%)
Missing	214 (12.7%)
**Have you received information regarding recommended exercises and physical activity after the planned treatment?**	
Yes	573 (34.0%)
No	957 (56.7%)
Missing	157 (9.3%)
**Have you received dietary recommendations to follow after the planned treatment?**	
Yes	374 (22.2%)
No	1146 (67.9%)
Missing	167 (9.9%)

The statistical assessment of the proportional odds assumption for the primary outcome models showed that the assumption held well for QoL levels 2–6. As very few patients reported QoL 0 or 1 at 1 year (3 and 13, respectively) the assumption could not be verified for these levels, and the results may not hold for those cases.

## Discussion

This study showed that most patients with colon cancer experienced a positive encounter with health care professionals. Apart from feeling well received, patients felt that they were taken seriously and that they were in safe hands. They also stated that they both dared and were offered the possibility to ask the questions they wanted to ask.

These results are consistent with a similar study in patients with rectal cancer in a similar context in Sweden and Denmark, (the QoliRECT cohort) [32]. Our study is in agreement with earlier qualitative studies reporting patients being treated and valued as unique persons, given the opportunity to ask questions and the perception of feeling in safe hands in order to establish a positive caring encounter [11, 14, 17, 18, 33].

Even though some patients felt that the waiting times were unreasonable it did not seem to affect their overall experience. Previous research has suggested that a positive experience of communication with health care professionals could alleviate the negative effect of long waiting times [16]. Still, it is difficult to elucidate from our study the patient experience during the actual waiting time and perhaps this is relevant to study in the future.

We hypothesized that a negative experience of care encounters was associated with low QoL. Due to the very limited number of reported negative experiences, no significant association could be related to QoL. Still, we found that several other factors were associated with QoL at 1 year. As previously shown in patients newly diagnosed with rectal cancer, we found that low pretreatment QoL, daily negative intrusive thoughts and low sense of coherence were associated with low QoL at follow up [28]. A Norwegian study found that a lower sense of coherence was associated with high age, physical function limitations and the need for home care nursing, among colorectal cancer survivors. The authors suggested that the level of sense of coherence ought to be considered to facilitate the optimal recovery and follow-up care [34]. It is possible that screening for sense of coherence and adjusting supportive care accordingly could improve QoL.

We also found that comorbidity, defined as a higher ASA-classification, was associated with QoL. This is not unexpected as poor physical status may be associated with low QoL. It is possible that supportive measures and rehabilitation could also have an effect on QoL. Another strong factor associated with low QoL was cancer recurrence. This was an expected result and highlights the importance of continued support of the patients throughout treatment and course of disease.

The patients reported feeling well informed about the disease and the planned treatment, but they did not recall having received what we consider to be clinically relevant information regarding potential functional side-effects. It is not possible to determine if the information was never given or if the information could not be perceived. We tried to reduce recall bias by administering the questionnaire within 1 week of the visit, so it is possible that the patients did not retain information. Regardless of if the information was insufficient or not perceived it did not seem to affect the feeling of being well informed. Similar results have been found previously among patients with rectal cancer [32]. Several studies have shown the importance of patients being sufficiently informed to cope and feel involved in the care and treatment process [11, 13, 14, 18]. However, there are challenges associated with providing information. The amount of information required, and when it should be given, is individual and changes over time [11, 14, 33, 35]. This confirms the importance of a person-centred approach to help the patient untangle what information is important.

Strengths of this study include the external and internal validity through control of the entire possible cohort. The large number of patients included and the multicentre design with 21 county and university hospitals are also strengths. Furthermore, the distribution of questionnaires by a neutral third party reduces bias [20].

Limitations of this study include possible selection bias. Patients who did not respond to the questionnaires had slightly more advanced tumours and higher comorbidity however this risk is mitigated by the overall high response rate. Another limitation is the choice to use a questionnaire that has been constructed for this study, rendering comparisons difficult. The questionnaire contains several questions covering different aspects of patient-reported experience of health care. The choice to use one of these questions as primary outcome could be considered a limitation, since single-item scales are prone to error. But we have also tested the other questions as secondary outcomes to explore any association with QoL, which enables a differentiated discussion of the experience of encounter with health care.

In conclusion this study showed that patients with colon cancer generally had a positive experience of the encounter with health care where they felt both well received and well informed. However, the amount of relevant information received was scarce. The results highlight the need to further explore this question. It also indicates that it may be difficult to identify whether patients are properly informed prior to treatment for colon cancer only by asking if they feel well informed. It is difficult for patients to assess whether they have been well-informed since very few know what clinically relevant information they are supposed to receive. Whether addressing sense of coherence or presence of negative intrusive thoughts at diagnosis could lead to improved QoL remains to be shown but is an interesting prospect.

## Supplementary Material

Patient reported experiences of health care, quality of life and preoperative information in colon cancer

## Data Availability

The participants of this study did not give written consent for their data to be shared publicly, so due to the sensitive nature of the research, supporting data are not available.
