# Supplementary material for: Patient reported experiences of health care, quality of life and preoperative information in colon cancer
Source: Acta Oncol. 2024 Nov 14;63:40933. doi: 10.2340/1651-226X.2024.40933 (PMC11586674; doi:10.2340/1651-226X.2024.40933)
Supplement: Patient reported experiences of health care, quality of life and preoperative information in colon cancer [file AO-63-40933-s1.pdf]

Supplementary material has been published as submitted. It has not been copyedited, or typeset by Acta Oncologica

Supplement table 1 Demography of non-responders and included patients

	Overall (N=1891)	Non-responders (N=204)	Included (N=1687)
**Sex**			
Male	977 (51.7%)	101 (49.5%)	876 (51.9%)
Female	914 (48.3%)	103 (50.5%)	811 (48.1%)
**Age (years)**			
Mean (SD)	71 (10)	73 (11)	71 (10)
**Tumor stage cT**			
T1-2	491 (26.0%)	43 (21.1%)	448 (26.6%)
T3	734 (38.8%)	79 (38.7%)	655 (38.8%)
T4	319 (16.9%)	44 (21.6%)	275 (16.3%)
TX	298 (15.8%)	33 (16.2%)	265 (15.7%)
Missing	49 (2.6%)	5 (2.5%)	44 (2.6%)
**Tumor stage cN**			
N0	976 (51.6%)	104 (51.0%)	872 (51.7%)
N1-2	751 (39.7%)	74 (36.3%)	677 (40.1%)
NX	121 (6.4%)	23 (11.3%)	98 (5.8%)
Missing	43 (2.3%)	3 (1.5%)	40 (2.4%)
**Tumor stage cM**			
M0	1668 (88.2%)	175 (85.8%)	1493 (88.5%)
M1	178 (9.4%)	25 (12.3%)	153 (9.1%)
Missing	45 (2.4%)	4 (2.0%)	41 (2.4%)
**Comorbidity (ASA-classification)**			
1	205 (10.8%)	9 (4.4%)	196 (11.6%)
2	1016 (53.7%)	84 (41.2%)	932 (55.2%)
3	479 (25.3%)	72 (35.3%)	407 (24.1%)
4	35 (1.9%)	8 (3.9%)	27 (1.6%)
5	1 (0.1%)	0 (0%)	1 (0.1%)
Missing	155 (8.2%)	31 (15.2%)	124 (7.4%)
**Intention of treatment**			
Curative	1666 (88.1%)	170 (83.3%)	1496 (88.7%)
Palliative	13 (0.7%)	1 (0.5%)	12 (0.7%)
Unknown	212 (11.2%)	33 (16.2%)	179 (10.6%)
